# Bio-Inspired Artificial Intelligence with Natural Language Processing Based on Deceptive Content Detection in Social Networking

**DOI:** 10.3390/biomimetics8060449

**Published:** 2023-09-23

**Authors:** Amani Abdulrahman Albraikan, Mohammed Maray, Faiz Abdullah Alotaibi, Mrim M. Alnfiai, Arun Kumar, Ahmed Sayed

**Affiliations:** 1 Department of Information Systems, College of Computer and Information Sciences, Princess Nourah Bint Abdulrahman University, P.O. Box 84428, Riyadh 11671, Saudi Arabia; 2 Department of Information Systems, College of Computer Science, King Khalid University, P.O. Box 394, Abha 61421, Saudi Arabia; 3 Department of Information science, College of Humanities and Social Sciences, King Saud University, P.O. Box 28095, Riyadh 11437, Saudi Arabia; 4 Department of Information Technology, College of Computers and Information Technology, Taif University, P.O. Box 11099, Taif 21944, Saudi Arabia; 5 Department of Electronics and Communication Engineering, New Horizon College of Engineering, Bengaluru 560103, India; 6 Research Center, Future University in Egypt, New Cairo 11835, Egypt

**Keywords:** social media, deceptive content detection, bio-inspired algorithm, African Vulture Optimization Algorithm, BiLSTM, artificial intelligence, deep learning

## Abstract

In recent research, fake news detection in social networking using Machine Learning (ML) and Deep Learning (DL) models has gained immense attention. The current research article presents the Bio-inspired Artificial Intelligence with Natural Language Processing Deceptive Content Detection (BAINLP-DCD) technique for social networking. The goal of the proposed BAINLP-DCD technique is to detect the presence of deceptive or fake content on social media. In order to accomplish this, the BAINLP-DCD algorithm applies data preprocessing to transform the input dataset into a meaningful format. For deceptive content detection, the BAINLP-DCD technique uses a Multi-Head Self-attention Bi-directional Long Short-Term Memory (MHS-BiLSTM) model. Finally, the African Vulture Optimization Algorithm (AVOA) is applied for the selection of optimum hyperparameters of the MHS-BiLSTM model. The proposed BAINLP-DCD algorithm was validated through simulation using two benchmark fake news datasets. The experimental outcomes portrayed the enhanced performance of the BAINLP-DCD technique, with maximum accuracy values of 92.19% and 92.56% on the BuzzFeed and PolitiFact datasets, respectively.

## 1. Introduction

In recent years, the popularity of social media has encouraged the spread of fake news like never before [1]. It is challenging to detect the fake news on social media as the false news is purposefully posted to mislead the public, leaving it challenging to distinguish the fake news from the original. So, in recent times, approaches to fake news recognition have captivated researchers [2]. In general, the framework for the detection of fake news related to social context encounters two difficulties in its modelling. The first issue is that the data in a social news context are heterogeneous and complex. Various kinds of entities are involved in social media content, for example, multiple types of connections, posts, replies, users, and re-posts. This creates following relations (user–user), responsive relations (post–post), and publishing relations (user–post) [3]. Though the heterogeneous features and connections of such an entity support the provision of evidence from diverse parties to confirm the news, a problem is their potential usage minus such evidence, with latter difficult to identify through modelling [4]. The second issue is the problem of distribution shift in modelling, where the training distribution varies with different test distributions.

Generally, the techniques for automatically identifying false data on social networking sites take advantage of social context data or news content [5]. Against this background, each dataset has its own strengths and weaknesses, and it is important not to work with incorrect data, which can create difficulties. Furthermore, despite the utility of social context data in enhancing the precision of approaches, various issues may cause substantial delays in fake news recognition [6]. These are both key considerations as the two most crucial factors in false data detection are early detection and accuracy [7]. Additionally, the highly adaptable nature of deceptive data plays a key role in obstructing the efficacy of the existing fake news recognition systems; here, a narrator can be useful to help ascertain the authenticity of news statements on a real-time basis. A further factor to consider is that it is hard to gain widespread data to train the systems for false news detection [8]. Moreover, it is a challenge to extract relevant attributes that can help in finding false news data in different fields [9]. Finally, it is difficult to find fake news about a new event as there are usually constrained data and knowledge concerning this event [10].

The current research article presents the Bio-inspired Artificial Intelligence with Natural Language Processing Deceptive Content Detection (BAINLP-DCD) technique in social networking. The goal of the BAINLP-DCD technique is to detect the presence of deceptive or fake content on social media. To accomplish this objective, the BAINLP-DCD algorithm uses a data preprocessing method to transform the input data into a meaningful format. For deceptive content detection, the BAINLP-DCD technique uses a Multi-Head Self-attention Bi-directional Long Short-Term Memory (MHS-BiLSTM) model. Finally, the African Vulture Optimization Algorithm (AVOA) is used for the selection of optimum hyperparameters for the MHS-BiLSTM model. The proposed BAINLP-DCD methodology was validated through simulation using two benchmark fake news datasets. The major contributions of the study are as follows.

- Development of a novel BAINLP-DCD technique encompassing MHS-BiLSTM-based classification and AVOA-based hyperparameter tuning for deceptive content detection. To the best of the authors’ knowledge, the proposed BAINLP-DCD technique is a new contribution to the literature.
- The parameter optimization of the MHS-BiLSTM model using the AVOA with cross-validation helps in boosting the predictive outcomes of the proposed model for unseen data.

## 2. Related Works

Nadeem et al. [11] introduced a hybrid technique named HyproBert for automated recognition of fake news. This technique utilizes DistilBERT for word embedding and tokenization. The embedding is fed as an input into the convolutional layer for extracting and highlighting the spatial features. Then, the output is presented to the Bidirectional Gated Recurrent Unit (BiGRU) for the extraction of the context features. Lastly, a dense layer is applied to integrate each feature for classification. In [12], the authors presented the Bidirectional Encoder Representation from Transformers (BERT)-related Deep Learning (DL) method by merging various parallel blocks of single-layer Deep Convolutional Neural Networks (DCNNs) and diverse kernel filters and sizes with BERT. Ahmad et al. [13] suggested new social-related and content-related attributes for the identification of rumors on social media networks. The study’s findings suggested that the presented features were highly useful in categorizing rumors compared with the existing baseline features. Likewise, the authors applied BiLSTM with a Recurrent Neural Network (Bi-LSTM-RNN) to text in order to detect rumors. This technique is a simple one, yet it has the potential to detect rumors. In the study, the experimentations on rumor detection were carried out using real-time datasets.

Kaliyar et al. [14] devised a deep CNN technique (FNDNet) for Fake News Detection (FND). Instead of depending on handcrafted features, the proposed method (FNDNet) was devised to discriminate between features automatically for the classification of false information using hidden layers, framed in the Deep Neural Network (DNN). The authors created a deep CNN to extract numerous features from all layers. Then, the study compared the performance of the presented method against various baseline approaches. In [15], a hyperparameter-tuned DL-related automatic FND (HDL-FND) method was proposed. This method accomplished results in terms of classification and detection of fake news. The proposed method encompassed a three-stage process involving BiLSTM-based classification, preprocessing, and feature extraction. Lee et al. [16] presented a DL structure for identifying fake news written in the Korean language. The earlier studies designed suitable FND methods for the English language, whereas the Korean language has two problems that cannot be overcome by the prevailing methods: first, it is hard for the DNN to function due to the feature scarcity for DL; second, due to morpheme ambiguity, semantic analysis is difficult.

Goldani et al. [17] modelled a CNN technique with margin loss and various embedding techniques for the detection of fake news. In this study, the static word embedding was compared against non-static embedding that offers the likelihood of gradually updating and up-training the word embeddings, during the training stage. The devised structure was assessed using two recent datasets in the domain, namely LIAR and ISOT. In [18], the authors carried out three experiments with Machine Learning (ML) methods, DL methods, and transformers. In all the experimentations, the authors relied upon the word embedding method for the extraction of contextual features. The experimental outcomes showed that the DL methods outpaced ML classifiers and BERT transformers in terms of accuracy. Additionally, the outcomes also displayed the same accuracy as those of the GRU and LSTM methods.

In [19], the performance of 16 meta-heuristic approaches in Artificial Neural Network (ANN) training was examined initially for the detection of non-linear systems. The study was designed to define the highly efficient meta-heuristic NN training systems. In [20], an NN-based method was presented for the detection of non-linear static methods. A modified algorithm named ABCES (ABC relies on Effective Scout Bee Stage) was established in this study for NN training. Via this approach, two essential alterations could be executed with ABCES. The authors of [21] developed a novel NN training approach named Hybrid Artificial Bee Colony algorithm based on the Effective Scout Bee Stage (HABCES). According to [22], the Feed-Forward NN (FFNN) training can be applied for Maximum Power Point Tracking (MPPT) by employing 13 Swarm-Intelligent (SI)-based optimizer systems. 

Most of the studies conducted already on deceptive content detection on social media were based on off-the-shelf ML or DL models with default hyperparameters. So, a need still exists to explore the influence of hyperparameter tuning on the performance of the model, especially in the case of deceptive content detection. Since social media data come in a huge volume and often contain noisy and unstructured content, it is challenging to detect the deceptive content. Hyperparameter-tuning techniques can help in optimizing the model architectures and hyperparameters to handle this complexity in an effective manner. Since trial-and-error hyperparameter tuning is a tedious process, in this work, the AVOA is applied for hyperparameter tuning.

## 3. The Proposed Model

**Figure 1:**
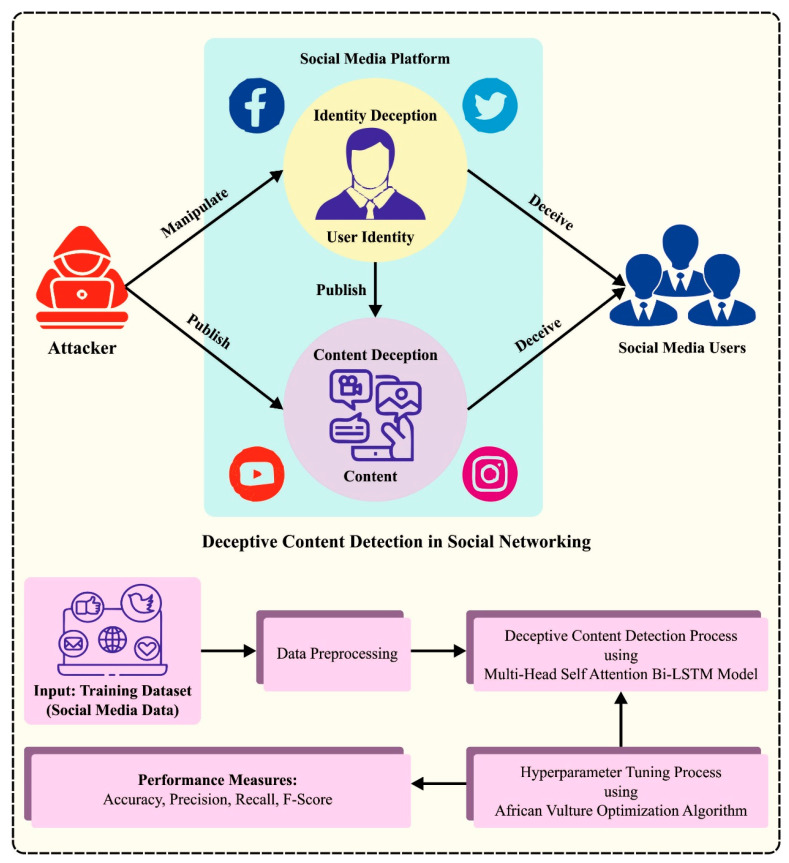
Overall procedure of the BAINLP-DCD method.

In the current study, we designed and developed the BAINLP-DCD technique for deceptive content detection in social media data. The major intention of the BAINLP-DCD technique is to detect the presence of deceptive or fake content in social media data. Figure 1 depicts the entire process of the proposed BAINLP-DCD approach. To accomplish its objective, the BAINLP-DCD technique follows a sequence of procedures such as data preprocessing, MHS-BiLSTM-based classification, and AVOA-based hyperparameter tuning. Initially, the input data are preprocessed to transform those into a useful format. Next, the detection process is conducted using the MHS-BiLSTM model. Finally, the AVOA is applied for the selection of the optimal hyperparameters for the MHS-BiLSTM model. 

### 3.1. Data Preprocessing

In general, the input data are preprocessed in different ways to improve the quality of the data. The outcomes from the data-mining process depend on a lot of preprocessing [23]. This adapts the unreliable and lacking raw data into representative machine-readable data. To accomplish this task, NLP methods like character alteration to lowercase letters, stemming, tokenization, stop word elimination, and other such processes in the Keras library are employed. Words such as ‘the,’ ‘of,’ ‘there,’ etc., are called stop words and are the most frequently utilized words in day-to-day conversations. As such, a stop word may appear in a text several times, though it likely has a limited impact in terms of the whole contexts of phrases. 

### 3.2. Detection Using the MHS-BiLSTM Model

For deceptive content detection, the BAINLP-DCD technique uses the MHS-BiLSTM model. LSTM-NN is a kind of RNN that presents a ‘gate’ model. It is capable of capturing the long-term semantic dependence and preventing the gradient-disappearing problems of the classical RNN, due to its long sequence [24]. Thus, the LSTM approach is applied for sentiment classification tasks. The computing method of the LSTM method is shown Equations (1)–(6).

(1) $$i_{t}=\sigma \left(W_{i}x_{t}+U_{i}h_{t-1}+V_{i}c_{t-1}\right)\;\;$$

(2) $${\tilde{C}}_{t}=tanh\left(W_{c}x_{t}+U_{c}h_{t-1}\right)\;\;$$

(3) $$f_{t}=\sigma \left(W_{f}x_{t}+U_{f}h_{t-1}+V_{f}c_{t-1}\right)$$

(4) $$c_{t}=f_{t}⊙c_{t-1}+i_{t}⊙{\tilde{C}}_{t}$$

(5) $$o_{t}=\sigma \left(W_{o}x_{t}+U_{o}h_{t-1}+V_{o}c_{t}\right)$$

(6) $$h_{t}=o_{t}⊙tanh\left(C\right)$$

Here, the input at t time, the cell layer, the value of the forget gate, the value of the input gate, the value of the output gate, the layer of the candidate cell, and the outcome of the LSTM units are represented by ${\;x}_{t},$ $c_{t},$ $i_{t},{\;f}_{t},$ $o_{t},$ ${\tilde{C}}_{t},$ and $h_{t},$ respectively. The sigmoid activation function is represented by σ, while ⊙ indicates the dot product function among the weighted matrices and $\{W_{*},U_{*},V_{*}{\}}_{*\in \{i,f,c,0\}}$ shows the parameter set from the LSTM unit. Hence, the BLSTM-NN technique is commonly utilized as the building block of the DL sentiment classification method to attain a better classification efficiency. 

Word2vec translates the text data of the assessment information into a vector representation that is fed as an input into the BLSTM. The final result of the sample sentiment classification is attained by passing the BLSTM to the sigmoid layer. 

Considering the sentence S, a pretrained model and a standard tokenizer are used to attain D dimensional embedding for a single word in the sentence, where $S=\left\{e_{1},e_{2},\ldots ,{\;e}_{N}\right\}$ and $S\in R^{N\times D}$ from the input to the model [25]. It is necessary to recognize certain words to identify the sarcasm in sentence S that provide relevant clues like negative emotions and sarcastic connotations. The importance of such a cue word corresponds to various factors based on dissimilar contexts. In this work, Multi-Head Self-Attention (MHSA) is leveraged to detect the cue words from the input texts.

Here, the attention module is used to determine the design in the input that is critical for determining the presented task. The self-attention model helps in learning a task’s particular connection amongst various modules to generate the best series representation. In this self-attention model, three linear projections exist, where K, Q, $V\in R^{N\times D},$ Key (K), Value (V), and Query (Q) of the given input order are created. The attention map is calculated according to the comparison among K, Q, and the outcomes of these modules. $A\in R^{N\times D}$ denotes the scaled Dot-product between V and learned softmax attention $\left(QK^{T}\right)$ as explained in Equation (7) as follows.

(7) $$A=softmax\left(\frac{QK^{T}}{\sqrt{D}}\right)V\;$$

**Figure 2:**
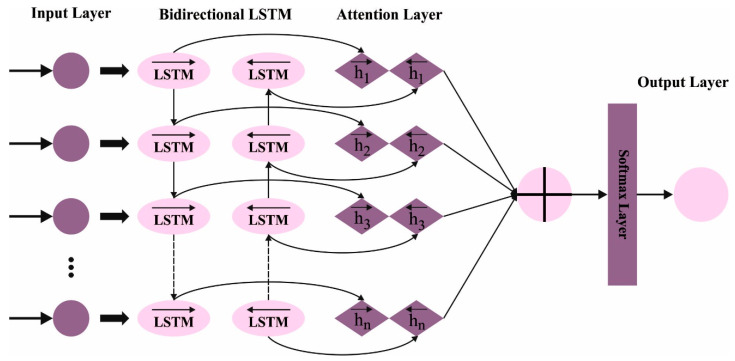
Architecture of attention BiLSTM.

Various copies of the self-attention model are utilized in parallel to MHSA. Every head captures the dissimilar connections among the individual keywords that support classification and identifies the words from the input text. In this work containing different heads $\left(\#H\right)$ in all the layers, a sequence of MHSA layers $\left(\#L\right)$ is used. Figure 2 demonstrates the framework of the attention BiLSTM layer.

### 3.3. Hyperparameter Tuning Using AVOA

In the current study, the AVOA is used for optimal fine-tuning of the hyperparameters for the MHS-BiLSTM model. AVOA is a new meta-heuristic swarm-based optimization approach inspired by the hunting style of the African vulture [26]. The African vulture is a kind of hunter that preys upon weak animals as its food. The AVOA is particularly inspired by its feeding and orienting behaviors. The algorithm consists of powerful operators while it also maintains a balance of exploration and efficiency in solving the continuous optimization problems.

In this method, there is an N count of the population of vultures and its values are adjusted to suit the problems that need to be resolved. The fitness of the vultures is measured after its arbitrary initialization. The best vulture is the vulture with the optimum solution, chosen to lead the first group; the second-best vulture is the vulture with next best solution, chosen to lead the rest of the groups. The remaining population is disseminated to make up both the groups, as in Equation (8). By applying the roulette wheel mechanism, the probability of choosing the group is calculated. The α and β parameters are predefined parameters so that the value lies in the range of [0, 1], where their sum is equivalent to 1. When α is closer to 1 and β lies near 0, the intensification increases in AVOA. If β is closer to 1 and α is closer to 0, this increases the diversification. The following equation provides more details in this regard.

(8) $$R_{i}=\left\{\begin{array}{l} BestVulTure_{1}\;if\;p_{i}=\alpha \\ BestVulTure_{2}\;if\;p_{i}=\beta \end{array}\right.$$

The starved vulture becomes aggressive; this stage defines the starvation rate of the vulture. A satiated vulture has abundant energy to travel a long distance, foraging for food; when it becomes starved, it becomes highly aggressive and finds food near other vultures. Equation (9) computes the satiation rate, which switches between the exploration and exploitation stages. Equation (10) ensures that the exploration stage reaches the exact estimate of the overall optimal solution and also that no early convergence takes place.

(9) $$F=\left(2\times rand+1\right)\times z\times \left(1-\frac{i}{i_{max}}\right)+t$$

(10) $$t=h\times \left({sin}^{\gamma }\left(\frac{\pi }{2}\times \frac{i}{i_{max}}\right)+cos\left(\frac{\pi }{2}\times \frac{i}{i_{max}}\right)-1\right)$$

Here, F is the rate of starvation, i represents the existing iteration, $i_{max}$ shows the max iteration count, and z indicates a random integer within [−1,1]; if z is negative, the vulture is starved, and if positive, the vulture is satisfied. rand denotes a random integer in [0,1] and h shows the random value within [−2,2]. The last iteration of the AVOA performs the exploitation and exploration stages.

The transformation between the exploration and exploitation phases can be accomplished by shifting the probability of entering the exploration stage at the last stage. If the parameter γ reduces, then the probability of turning towards the exploration phase in the end stage reduces. At last, if $\left|F\right|$ is less than 1, then the vulture is starved and finds prey in the following region, and accordingly, the AVOA enters the exploitation stage. If $\left|F\right|$ exceeds one, then the vulture is satisfied and pursues food foraging in other regions. Therefore, the AVOA enters the exploration stage. 

In the exploration stage, the vulture can find food and travel for a long distance. The vulture identifies a new area based on the satiation level, and a parameter $P_{1}$ is used for the selection process, as shown in the Equations (11) and (12). Two strategies are used to ensure a wider exploration of the search range. The initial strategy considers the exploration of the search region, adjacent to a better vulture from the group. This technique enables the localized exploration from the neighborhood of the present optimum performance. On the other hand, the next approach allows exploration across the whole search range while following the specific upper and lower limitations. This method permits a wider exploration range without surpassing the limits.

(11) $$P\left(i+1\right)=R\left(i\right)-D\left(i\right)\times F\;D\left(i\right)=\left|X\times R\left(i\right)-P\left(i\right)\right|P_{1}\geq rand_{P1}\;\;$$

(12) $$P\left(i+1\right)=R\left(i\right)-F+rand_{2}\times \left(\left(ub-lb\right)\times rand_{3}+lb\right)\;\;\;P_{1}<rand_{P1}$$

Here$,\;P_{1}$ denotes the random integer that lies in the range of [0,1], R(i) indicates the $vulture_{-}i$ leader, and F shows the rate of starvation, as set out in (9). X co-efficient is used for increasing the arbitrary movement and alteration with all the iterations. ub and lb indicate the upper and lower boundaries, respectively. $rand_{P1}$, $rand_{2}$, and $rand_{3}$ are random integers within [0, 1].

When the rate of starvation is |F|<l, then it enters the exploitation stage, which has two phases. If the values of the rate of starvation range between 1 and 0.5, then the AVOA technique enters the first exploitation stage. However, the vulture remains comparatively satisfied. At this point, a random number $\left(rand_{P2}\right)$ is created in [0, 1] for choosing that specific approach to follow, and later, it is compared to a predetermined parameter $P_{2}$. The siege fight strategy is applied if $rand_{P2}$ is equal to or greater than $P_{2}$. In this strategy, the vulture behaviors are simulated, i.e., when a strong vulture refuses to share food, the weaker vultures tend to tire this strong vulture out by surrounding and attacking it. The rotating flight is chosen if $rand_{P2}$ is less than $P_{2},$ i.e., spiral motion of a vulture while finding food. Equations (13) and (14) represent the siege and rotation flights, respectively.

(13) $$P\left(i+1\right)=D\left(i\right)\times \left(F+rand_{4}\right)-d\left(t\right)\;d\left(t\right)=R\left(i\right)-P\left(i\right)\;P_{2}\geq rand_{P2}\;$$

(14) $$P\left(i+1\right)=R(i)-(S_{1}+S_{2})\;S_{1}=R(i)\times \left(\frac{rand_{5}\times P\left(i\right)}{2\pi }\right)\times cos\left(P\left(i\right)\right)\;S_{2}=R\left(i\right)\times \left(\frac{rand_{6}\times P\left(i\right)}{2\pi }\right)\times sin\left(P\left(i\right)\right)\;P_{2}<rand_{P2}$$

Here $D\left(i\right)$ is evaluated in (10), F denotes the rate of starvation, R(i) indicates the vulture’s leader, P(i) stands for the present location of the vultures, and $rand_{4}$, $rand_{P2}$ and $rand_{6}$ denote the random numbers within [0, 1], which, in turn, increase the arbitrariness. The values of sin(P(i)) or $cos\left(P\left(i\right)\right)$ form an array vector of dimension n×1, whereas n indicates the count of units generated.

If |F|<0.5, the vulture is starved and aggressive in the second exploitation phase. Firstly, a random integer $rand_{P3}$ within [0, 1] is compared to a predetermined parameter $P_{3}$. When $rand_{P3}$ is equal to or greater than $P_{3}$, different kinds of vultures accumulate over the food. In other terms, an aggressive siege fight is selected as follows.

(15) $$P\left(i+1\right)=\frac{A_{1}+A_{2}}{2}\;A_{1}=BestVulture_{1}\left(i\right)-\frac{BestVulture_{1}\left(i\right)\times P\left(i\right)}{BestVulture_{1}(i)\times P(i{)}^{2}}\times F\;P_{3}\geq rand_{P3}$$

(16) $$A_{2}=BestVulture_{2}(i)-\frac{BestVulture_{2}\left(i\right)\times P\left(i\right)}{BestVulture_{2}\left(i\right)\times P(i{)}^{2}}\times F\;P\left(i+1\right)=R\left(i\right)-\left|d\left(t\right)\right|\times F\times Levy\left(d\right)\;P_{3}<rand_{P3}$$

Here, the first and the second best vultures in the existing iteration are represented as $BestVulture_{1}(i)$ and $BestVulture_{2}\left(i\right),$ respectively. d is the problem dimension. P(i) denotes the existing location of the vultures. R(i), d(t), and F were described earlier, while the levy flight increases the efficacy of the AVOA and is evaluated as follows.

(17) $$LF\left(x\right)=0.01\times \frac{u\times \sigma }{1}\;\sigma ={\left(\frac{\Gamma \left(1+\beta \right)\times \sin\left(\frac{\pi \beta }{2}\right)}{\Gamma \left(1+\beta 2\right)\times \beta \times 2\left(\frac{\beta -1}{2}\right)}\right)}^{\frac{1}{\beta }}\;\;$$

In Equation (17), β is a set number that is equivalent to 1.5, whereas u and v are randomly generated values within [0, 1].

The AVOA system progresses a Fitness Function (FF) to realize the best classifier solution. It defines a positive integer to denote the best solution for candidate efficiencies. In this case, FF corresponds to the reduction in classifier errors, as expressed in Equation (18).

(18) $$fitness\left(x_{i}\right)=Classifier\;Error\;Rate\left(x_{i}\right)\;=\frac{No.\;of\;misclassified\;instances\;}{Total\;no.\;of\;instances\;}*100$$

## 4. Experimental Validation

The proposed model was simulated using the Python 3.6.5 tool on a PC configured with i5-8600k, GeForce 1050Ti 4 GB, 16 GB RAM, 250 GB SSD, and 1 TB HDD. The parameter settings used for the investigation were as follows: learning rate: 0.01, dropout: 0.5, batch size: 5, epoch count: 50, and activation: ReLU.

The experimentation analysis for the BAINLP-DCD method was conducted using two datasets, Buzz Feed and PolitiFact. Table 1 shows the details of the BuzzFeed dataset [27].

**Figure 3:**
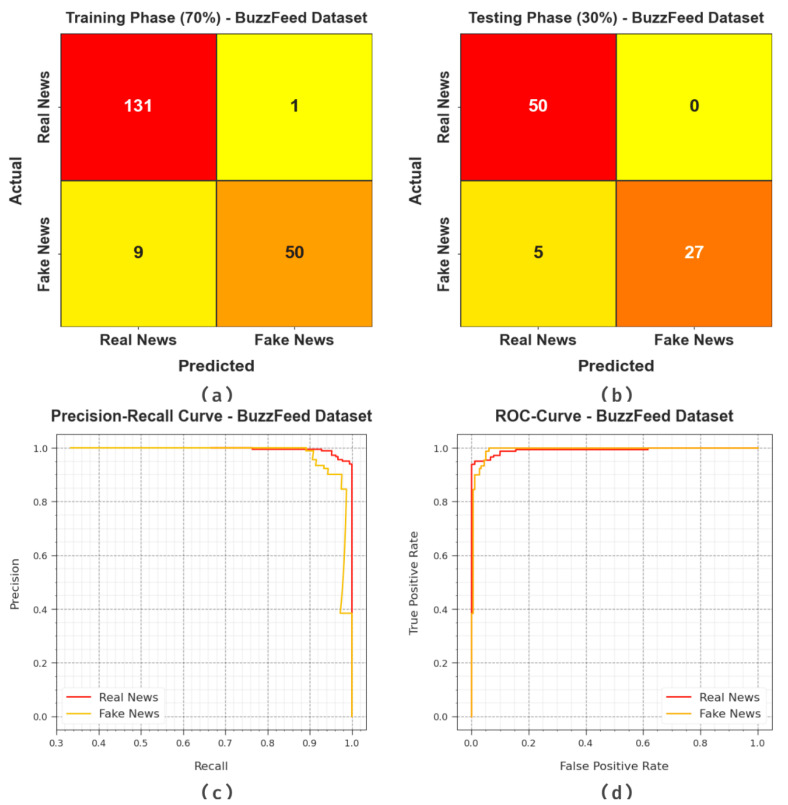
Classification results on BuzzFeed dataset.

Figure 3 illustrates the results accomplished by the BAINLP-DCD method on the BuzzFeed dataset. Figure 3a,b show the confusion matrices generated with the BAINLP-DCD technique at a 70:30 ratio of training phase (TRP)/testing phase (TSP). The figure signifies that the BAINLP-DCD approach identified and classified both the classes accurately. Also, Figure 3c exhibits the PR curve of the BAINLP-DCD algorithm. The figure denotes that the BAINLP-DCD method attained the optimum Precision-Recall (PR) performance on two class labels. Finally, Figure 3d demonstrates the ROC of the BAINLP-DCD method. The figure reveals that the BAINLP-DCD system produced effective results with higher ROC values under both the class labels.

**Figure 4:**
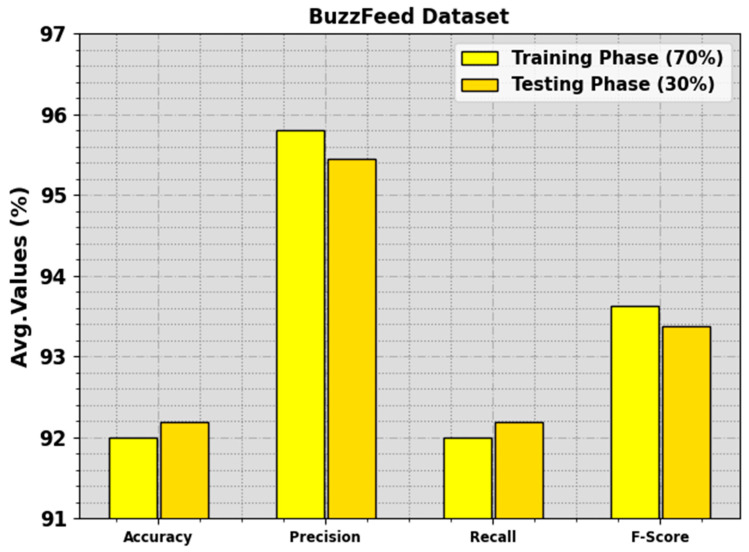
Average values of the BAINLP-DCD technique on the BuzzFeed dataset.

The fake news detection results attained by the BAINLP-DCD technique on the BuzzFeed dataset are shown in Table 2 and Figure 4. The outcomes show that the BAINLP-DCD system properly categorized the real news class from the fake news. On the 70% TR set, the BAINLP-DCD technique gained average $accu_{y}$, $prec_{n}$, $reca_{l}$, and $F_{score}$ values of 91.99%, 95.81%, 91.99%, and 93.62%, respectively. In addition to this, on the 30% TS set, the BAINLP-DCD system achieved average $accu_{y}$, $prec_{n}$, $reca_{l}$, and $F_{score}$ values of 92.19%, 95.45%, 92.19%, and 93.38%, respectively.

**Figure 5:**
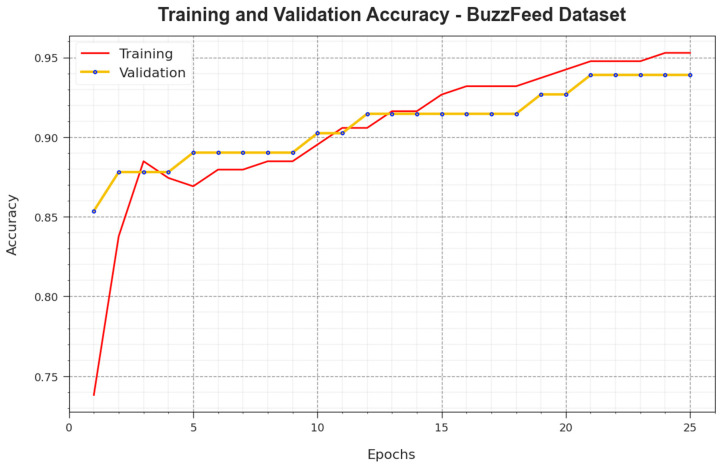
$Accu_{y}$ curve of the BAINLP-DCD technique on the BuzzFeed dataset.

Figure 5 demonstrates the training accuracy curves $\;TR\_accu_{y}$ and $VL\_accu_{y}$ accomplished by the BAINLP-DCD technique on the BuzzFeed dataset. $TL\_accu_{y}$ was determined by evaluating the BAINLP-DCD system on the TR dataset, whereas $VL\_accu_{y}$ was calculated by evaluating the performance on separate testing datasets. The experimental outcomes infer that the $TR\_accu_{y}$ and $VL\_accu_{y}$ values increase with an upsurge in the number of epochs. Therefore, the performance of the BAINLP-DCD method was enhanced on the TR and TS datasets with an increase in the number of epochs.

**Figure 6:**
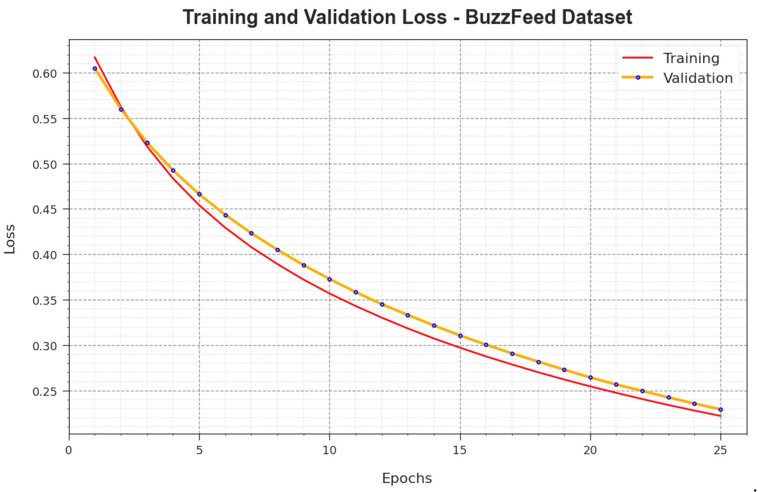
Loss curve of the BAINLP-DCD technique on the BuzzFeed dataset.

In Figure 6, the TR_loss and VR_loss analysis values achieved with the BAINLP-DCD method on the BuzzFeed dataset are presented. The TR_loss is determined as the error between the predicted performance and original values of the TR data. The VR_loss denotes a measure of performance of the BAINLP-DCD approach on separate validation data. The outcomes specify that the TR_loss and VR_loss values tend to lessen with a greater number of epochs. The outcomes establish the improved performance of the BAINLP-DCD system and its ability to accomplish accurate classification. The decreased TR_loss and VR_loss values show the better performance of the BAINLP-DCD approach in capturing the relationships and patterns.

**Figure 7:**
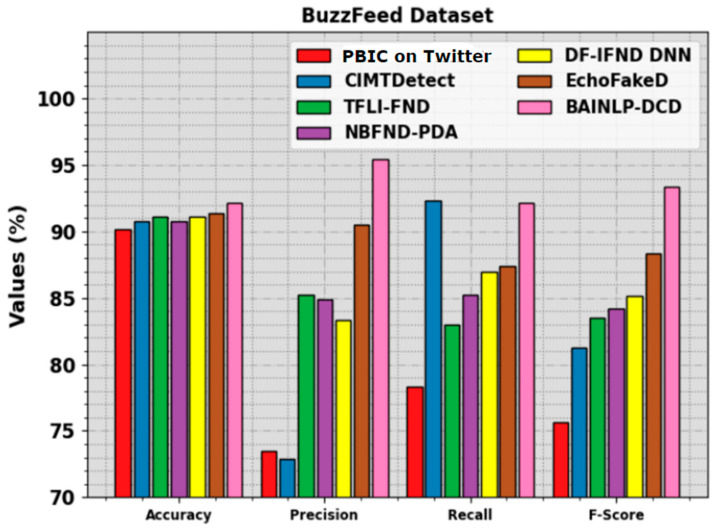
Comparative outcomes of the BAINLP-DCD algorithm on BuzzFeed dataset.

In Table 3 and Figure 7, the comparative analysis outcomes of the BAINLP-DCD system on the BuzzFeed dataset are portrayed [1]. The experimental outcomes denote that the PBIC, CIMTDetect, and NBFND-PDA techniques accomplished poor performance. Simultaneously, the TFLI-FND, DF-IFND DNN, and EchoFakeD algorithm achieved closer outcomes. However, the BAINLP-DCD method achieves a superior performance with $accu_{y}$, $prec_{n}$, $reca_{l}$, and $F_{score}$ values of 92.19%, 95.45%, 92.19%, and 93.38%, respectively.

Table 4 provides a detailed description of the PolitiFact dataset.

**Figure 8:**
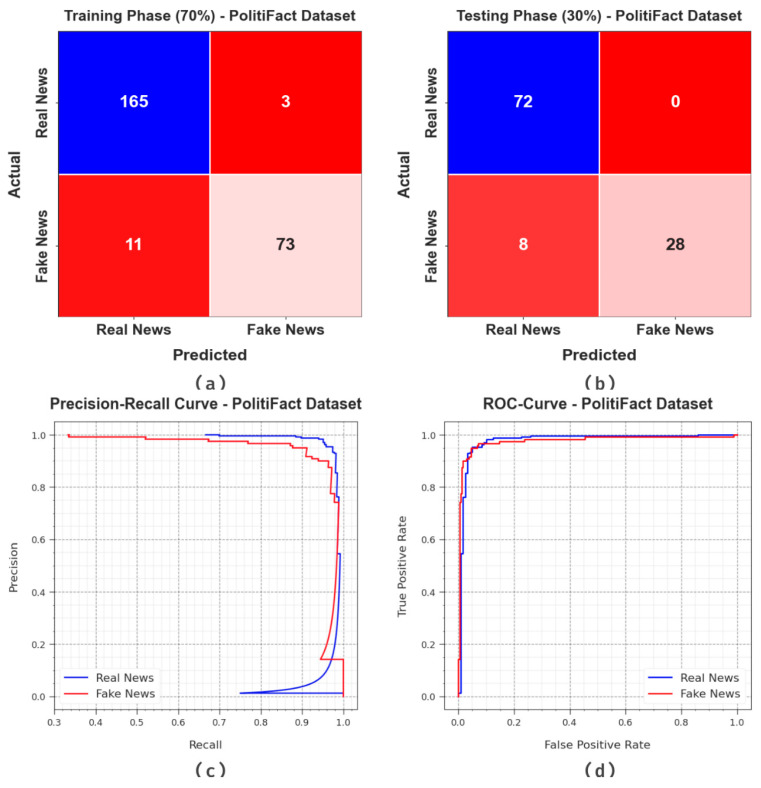
Classification results on PolitiFact dataset.

Figure 8 portrays the classifier analysis outcomes of the BAINLP-DCD method on the PolitiFact dataset. Figure 8a,b show the confusion matrices generated by the BAINLP-DCD technique on a 70:30 TRP/TSP dataset. The outcomes signify that the BAINLP-DCD approach recognized both the classes precisely. Also, Figure 8c shows the PR curve of the BAINLP-DCD model. The figure denotes that the BAINLP-DCD system attained the maximum PR values under both the class labels. Finally, Figure 8d demonstrates the ROC of the BAINLP-DCD system. The figure depicts that the BAINLP-DCD method achieved efficient outcomes with higher ROC values on the two class labels.

**Figure 9:**
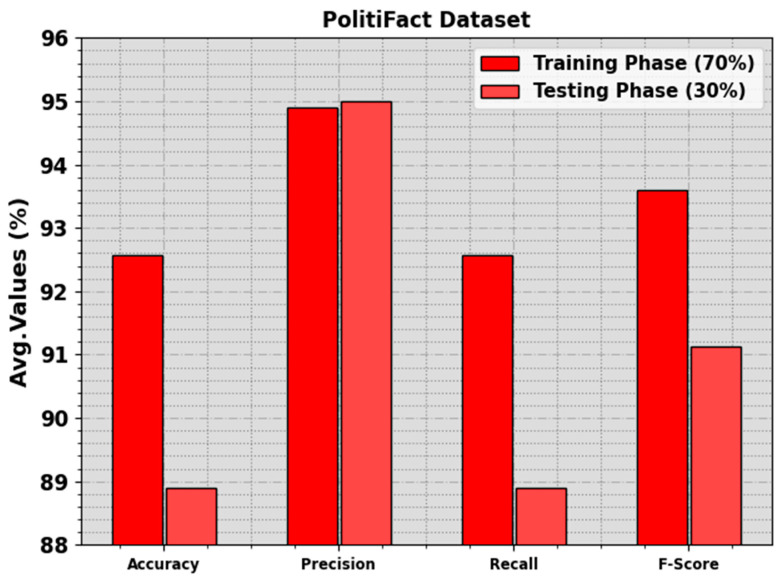
Average values of the BAINLP-DCD technique on PolitiFact dataset.

The fake news detection outcomes of the BAINLP-DCD system on the PolitiFact dataset are shown in Table 5 and Figure 9. These values indicate that the BAINLP-DCD method appropriately categorized the real and fake news class labels. On the 70% TR set, the BAINLP-DCD approach achieved an increase in the average $accu_{y}$, $prec_{n}$, $reca_{l}$, and $F_{score}$ values, with 92.56%, 94.90%, 92.56%, and 93.59%, respectively. Additionally, on the 30% TS set, the BAINLP-DCD system gained average $accu_{y}$, $prec_{n}$, $reca_{l}$, and $F_{score}$ values of 88.89%, 95%, 88.89%, and 91.12%, respectively.

**Figure 10:**
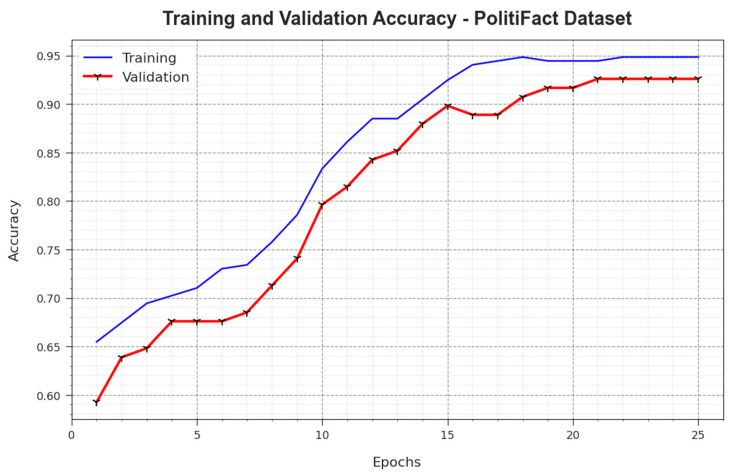
$Accu_{y}$ curve of the BAINLP-DCD technique on the PolitiFact dataset.

Figure 10 illustrates the training accuracy, i.e., $TR\_accu_{y}$ and $VL\_accu_{y}$ values achieved by the BAINLP-DCD system on the PolitiFact dataset. $TL\_accu_{y}$ is determined by estimated the BAINLP-DCD algorithm on TR datasets, whereas $VL\_accu_{y}$ is calculated by evaluating the performance on a separate testing dataset. The investigational results show that the $TR\_accu_{y}$ and $VL\_accu_{y}$ values increase with an upsurge in the number of epochs. Thus, the performance of the BAINLP-DCD approach achieved enhanced accuracy values on both TR and TS datasets with an increase in the number of epochs.

**Figure 11:**
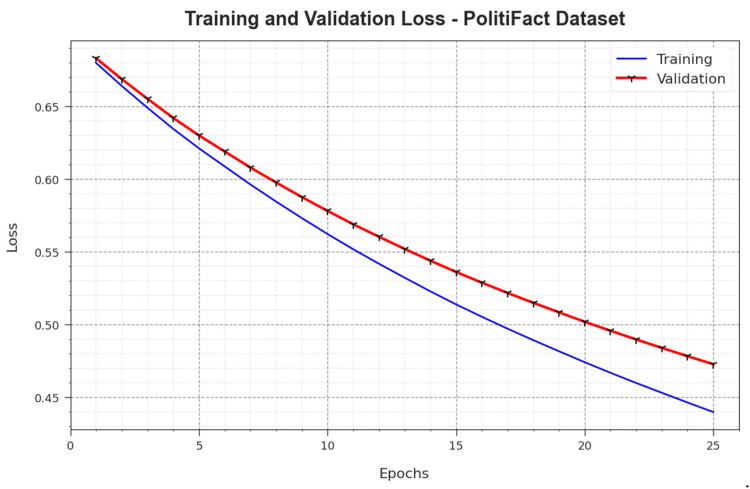
Loss curve of the BAINLP-DCD technique on the PolitiFact dataset.

In Figure 11, the TR_loss and VR_loss analysis outcomes achieved by the BAINLP-DCD system on the PolitiFact dataset are demonstrated. TR_loss defines the error between the predicted performance and the original values on the TR data, while VR_loss signifies a measure of performance for the proposed BAINLP-DCD system on a separate validation dataset. The experimental outcomes denote that the TR_loss and VR_loss values tend to reduce with an increase in the number of epochs. These outcomes depict the enriched performance of the BAINLP-DCD method and its ability to accomplish accurate classification. The minimized TR_loss and VR_loss values confirm the superior performance of the BAINLP-DCD approach in capturing the relationships and patterns.

**Figure 12:**
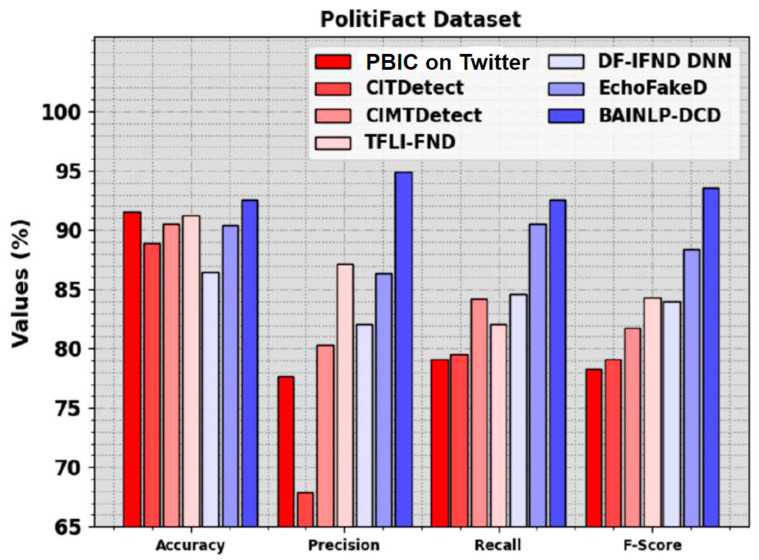
Comparative outcomes of the BAINLP-DCD algorithm and other recent models on PolitiFact dataset.

In Table 6 and Figure 12, the comparison study outcomes of the BAINLP-DCD technique and other techniques using the PolitiFact dataset are given. The results show that the PBIC, CIMTDetect, and CITDetect models accomplished the worst performance. At the same time, the TFLI-FND, DF-IFND DNN, and EchoFakeD models attain closer results. However, the BAINLP-DCD technique reaches the maximum performance with $accu_{y}$, $prec_{n}$, $reca_{l}$, and $F_{score}$ values of 92.56%, 94.90%, 92.56%, and 93.59%, respectively.

Therefore, the proposed BAINLP-DCD system can be applied in automated fake news detection. The enhanced performance of the BAINLP-DCD technique is due to the inclusion of an AVOA-based hyperparameter-tuning process. Hyperparameters are settings that should be set prior to the training process as they are not learned during the training process. They exert a significant impact on the performance of the model, and selection of the optimal values can lead to better accuracy. By applying AVOA-based hyperparameter tuning, the proposed model achieves better results thanks to the selection of the optimal settings for the algorithm. These results confirm the improved performance of the proposed technique over other existing techniques.

## 5. Conclusions and Future Work

In the current study, we designed and developed the BAINLP-DCD technique for deceptive content detection in social media data. The major intention of the BAINLP-DCD technique is to detect the presence of deceptive or fake content in social media data. In order to accomplish this objective, the BAINLP-DCD technique follows a sequence of procedures like data preprocessing, MHS-BiLSTM-based classification, and AVOD-based hyperparameter tuning. For deceptive content detection, the BAINLP-DCD technique uses the MHS-BiLSTM model. Finally, the AVOA technique is applied for optimal hyperparameter selection of the MHS-BiLSTM network. The proposed BAINLP-DCD system was experimentally validated through simulation using two fake news datasets. The experimental outcomes confirmed the enhanced performance of the BAINLP-DCD method, with maximum accuracy values of 92.19% and 92.56% on the BuzzFeed and PolitiFact datasets, respectively. 

In the future, the proposed model can be extended for sarcasm detection and classification processes. Furthermore, future works can extend the proposed model to multi-class classification, as well. Fine-grained classification techniques can also be explored to classify the deceptive content into different classes, like misinformation, disinformation, fake news, propaganda, etc., and thus provide more nuanced insights into the nature of deceptive content. In addition, the explainability and interpretability of the DL approaches can also be explored for deceptive content detection. Moreover, the computational complexity of the proposed model can be examined in the future. 

## Figures and Tables

**Table 1:** Details of BuzzFeed dataset.

BuzzFeed Dataset	
Class	No. of Samples
Real News	182
Fake News	91
Total Samples	273

**Table 2:** Fake news detection outcomes when using the BAINLP-DCD technique on the BuzzFeed dataset.

Class	$Accu_{y}$	$Prec_{n}$	$Reca_{l}$	$F_{score}$
**Training Phase (70%)**				
Real News	99.24	93.57	99.24	96.32
Fake News	84.75	98.04	84.75	90.91
Average	91.99	95.81	91.99	93.62
**Testing Phase (30%)**				
Real News	100.00	90.91	100.00	95.24
Fake News	84.38	100.00	84.38	91.53
Average	92.19	95.45	92.19	93.38

**Table 3:** Comparison analysis outcomes of the BAINLP-DCD method and other recent systems on the BuzzFeed dataset.

BuzzFeed Dataset				
Methods	$Accu_{y}$	$Prec_{n}$	$Reca_{l}$	$F_{score}$
PBIC on Twitter	90.12	73.50	78.30	75.60
CIMTDetect	90.74	72.90	92.30	81.30
TFLI-FND	91.08	85.20	83.00	83.50
NBFND-PDA	90.80	84.90	85.20	84.20
DF-IFND DNN	91.09	83.33	86.96	85.11
EchoFakeD	91.38	90.47	87.36	88.37
BAINLP-DCD	92.19	95.45	92.19	93.38

**Table 4:** Details of PolitiFact dataset.

PolitiFact Dataset	
Class	No. of Samples
Real News	240
Fake News	120
Total Samples	360

**Table 5:** Fake news detection outcomes of the BAINLP-DCD technique on the PolitiFact dataset.

Class	$Accu_{y}$	$Prec_{n}$	$Reca_{l}$	$F_{score}$
**Training Phase (70%)**				
Real News	98.21	93.75	98.21	95.93
Fake News	86.90	96.05	86.90	91.25
Average	92.56	94.90	92.56	93.59
**Testing Phase (30%)**				
Real News	100.00	90.00	100.00	94.74
Fake News	77.78	100.00	77.78	87.50
Average	88.89	95.00	88.89	91.12

**Table 6:** Comparison study outcomes of the BAINLP-DCD method and other recent methods on the PolitiFact dataset.

PolitiFact Dataset				
Methods	$Accu_{y}$	$Prec_{n}$	$Reca_{l}$	$F_{score}$
PBIC on Twitter	91.53	77.70	79.10	78.30
CITDetect	88.85	67.90	97.50	79.10
CIMTDetect	90.50	80.30	84.20	81.80
TFLI-FND	91.26	87.20	82.10	84.30
DF-IFND DNN	86.39	82.10	84.60	84.04
EchoFakeD	90.43	86.36	90.48	88.37
BAINLP-DCD	92.56	94.90	92.56	93.59

## Data Availability

Data sharing does not apply to this article as no datasets were generated during the current study.
